# Autonomous control of an ultrasound probe for intra-operative ultrasonography using vision-based shape sensing of pneumatically attachable flexible rails

**DOI:** 10.1007/s11548-024-03178-z

**Published:** 2024-05-22

**Authors:** Aoife McDonald-Bowyer, Tom Syer, Adam Retter, Danail Stoyanov, Agostino Stilli

**Affiliations:** 1 WEISS, UCL, London, UK; 2 Department of Radiology, University of Cambridge, Cambridge, UK; 3 Centre for Medical Imaging, UCL, London, UK

**Keywords:** Medical robotics, Intra-operative ultrasound, Shape sensing, Robotic-assisted surgery

## Abstract

**Purpose:**

In robotic-assisted minimally invasive surgery, surgeons often use intra-operative ultrasound to visualise endophytic structures and localise resection margins. This must be performed by a highly skilled surgeon. Automating this subtask may reduce the cognitive load for the surgeon and improve patient outcomes.

**Methods:**

We demonstrate vision-based shape sensing of the pneumatically attachable flexible (PAF) rail by using colour-dependent image segmentation. The shape-sensing framework is evaluated on known curves ranging from $r = 30$ to $r = 110$ mm, replicating curvatures in a human kidney. The shape sensing is then used to inform path planning of a collaborative robot arm paired with an intra-operative ultrasound probe. We execute 15 autonomous ultrasound scans of a tumour-embedded kidney phantom and retrieve viable ultrasound images, as well as seven freehand ultrasound scans for comparison.

**Results:**

The vision-based sensor is shown to have comparable sensing accuracy with FBGS-based systems. We find the RMSE of the vision-based shape sensing of the PAF rail compared with ground truth to be $0.4975 \pm 0.4169$ mm. The ultrasound images acquired by the robot and by the human were evaluated by two independent clinicians. The median score across all criteria for both readers was ‘3—good’ for human and ‘4—very good’ for robot.

**Conclusion:**

We have proposed a framework for autonomous intra-operative US scanning using vision-based shape sensing to inform path planning. Ultrasound images were evaluated by clinicians for sharpness of image, clarity of structures visible, and contrast of solid and fluid areas. Clinicians evaluated that robot-acquired images were superior to human-acquired images in all metrics. Future work will translate the framework to a da Vinci surgical robot.

**Supplementary Information:**

The online version contains supplementary material available at 10.1007/s11548-024-03178-z.

## Introduction

Robotic-assisted minimally invasive surgery is now widely employed in hospitals worldwide as its benefits are extensive [1]. Namely, it shortens procedure time and hospital stays, reduces postoperative pain and minimises recovery time. The robotic approach also benefits surgeons; the console has an ergonomic design, meaning reduced discomfort and fatigue, while the increased dexterity of the robotic arms allows surgeons to operate on hard-to-reach areas. This gives the potential for far greater precision to achieve better resection margins and reduces the risk of damage to healthy areas [2]. However, robotic procedures often require years of specialist training[3].

The robotic approach is frequently employed in abdominal cancer resections [4], including robotic-assisted partial nephrectomy (RAPN) [5], as it facilitates the sparing of nephrons, improving long-term outcomes. However, this procedure remains one of the most demanding in urological surgery [3].

One of the difficult subtasks of these surgeries is identifying tumour margins with intra-operative imaging, particularly for endophytic subtypes [6]. Usually, a drop-in ultrasound (US) probe is introduced to the surgical scene, and the surface of the kidney is swiped multiple times to visualise the tumour lying beneath the surface [6].

Mobilising the kidney for tumour resection is a challenging task due to its complex anatomy, the perirenal fat layer typically present on its highly vascularised surface and the need to interpret preoperative images. The surgeon must simultaneously manipulate the kidney, interpret images and mark resection margins, a process prone to errors and excessively large margins.

In previous work, we have introduced a novel soft silicone-based device to assist with the US scanning subtask [7]. The pneumatically attachable flexible (PAF) rail is a soft mechanical interface that adheres to the surface of organs via pneumatic suction. The complete deployment process of the PAF rail and its use with US probes is detailed in [8]. We demonstrated how the PAF rail could acquire US images for 3D reconstruction in [10]. We also showed how shape and interaction information can be extracted by embedding a fibre-optic shape sensor into the body of the PAF rail in [9–11].

One of the purposes of the PAF rail is to provide a stable track for the US probe to attach to (via a custom attachment), limiting the range of motion to 2 degrees of freedom to reduce the cognitive load on the surgeon. It also provides an anchor for the US probe, meaning the surgeon is not solely responsible for keeping the probe in the surgical field. Concurrently, it allows the surgeon to execute the same trajectory repeatedly, which will improve US image quality for 3D volume reconstruction, with potential for augmented reality systems. Furthermore, automating this subtask could help reduce the steep learning curve associated with RAPN. While numerous studies have focused on investigating automated extracorporeal ultrasound scanning on the patient’s skin [12–14], intracorporeal or intra-operative ultrasound presents many challenges that are yet to be addressed [15, 16]. One such challenge is the registration of ultrasound images with spatial information to make them suitable for automating. Researchers have largely focused on optical tracking using fiducial markers [15]. Other approaches have considered electromagnetic tracking [16], but this is limited by metallic instruments and the need for a separate generator. Several studies have focused on creating hardware for this purpose [16]. More recently, [17] building on the work of [18] used a deep learning approach to ensure robust probe tissue on a convex surface, replicating the intra-operative scenario. Their approach was only tested on vessel segmentation, and while showed promising results, would require more experimentation and data to prove reliable for other anatomy and tissue types. A comprehensive review of robot-assisted US systems is found in [19].

Considering the PAF rail will already be in the operative field when deployed, we can exploit its presence in the endoscopic field of view. With little and inexpensive hardware modification, we propose using the PAF rail as a fiducial marker for vision-based tracking and extraction of information. In this work, we explore the exploitation of colour-based segmentation and feature extraction to extract shape information of the PAF rail. It is quick and inexpensive to modify the colour(s) of the PAF rail without altering the mechanical properties of the device. We hypothesise that from sensing the shape of the PAF rail, the shape of the organ it is attached to can be inferred. We can then use this information to inform path planning for a robot holding the US probe to execute an autonomous scan.

Vision-based shape-sensing methods for continuum and flexible robots’ have existed for some time [20, 21]. For soft robots, shape sensing often involves strain [22], optical [23] or electromagnetic sensors [24], which can undesirably affect their material properties and incur extra costs due to additional hardware.

Vision-based sensing using external cameras has effectively measured soft body deformations in tactile tasks by tracking fiducial markers like dot patterns [25, 26]. Such methods not only estimate contact force from material deformation but also provide an accurate approach for soft material shape sensing. Data-driven shape sensing using embedded cameras and CNNs for capturing deformation patterns has been explored [27]. Additionally, a fusion of FBG sensing with stereo vision has achieved precise shape sensing, even in cases of visual occlusion [28]. While FBG sensing has been successfully explored in the PAF rail [9], our application finds vision-based sensing more suitable due to its non-intrusive nature and the availability of stereo endoscopes in surgical settings.

In this paper, we propose a vision-based shape-sensing framework to guide autonomous robotic US scanning in surgeries. This system estimates the 2D shape of a flexible interface in real time, enabling a robot arm with a US probe to follow a planned trajectory for scanning, leveraging equipment already present in surgical environments.

**Fig. 1:**
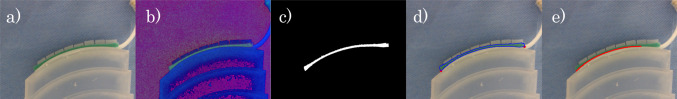
Process of the vision-based shape sensing of the PAF rail: **a** Input RGB image is calibrated. **b**)RGB image is converted to HSV colourspace **c** Image is thresholded based on user-selected colour, and a mask is generated. **d** A contour of the mask is found. Corners of the contour are detected. **e** A portion of the contour between the edges is selected and a spline approximation is applied to those points. The spline curve is downsampled to a 2 $\times$ 100 array and published to a ROS topic

## Materials and methods

### Design and fabrication of the PAF rail

**Fig. 2:**
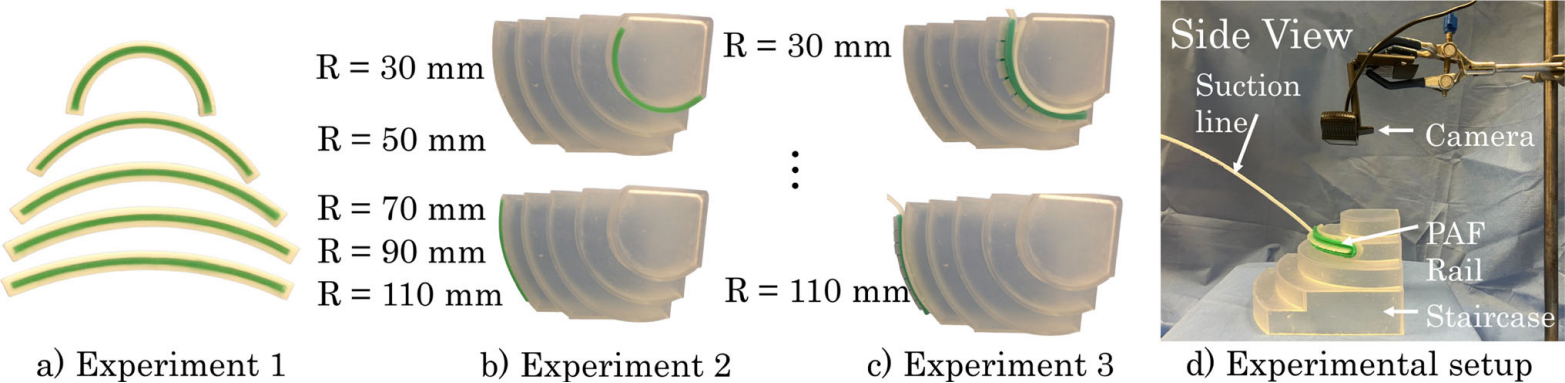
**a** First experiment on colourbands in their moulds. **b** Second experiment on colourbands placed on the staircase with profiles of radius ranging from 110 mm to 30 mm in 20 mm increments. **c** PAF rail suctioned to the profiles of each step of the staircase. **d** Experimental setup for the vision-based shape sensing

The design of the PAF rail suction line used in this work is based on the optimisation study first presented in [8, 27], and later iterations in [9, 11]. In this version, we wanted to manufacture the rail in solid block colours such that the suction area and grasping fin are in one colour, and the rail is in another contrasting colour, as shown in Fig. 2d. This was achieved using a two-step injection moulding process. We moulded rail prototypes in DragonSkin^TM^ 30 (Shore hardness 30 A) silicone elastomer (Smooth-On Inc., Macungie, PA, US) with added green pigmentation.

### Colour-based shape estimation

The colour-based shape estimation was performed using the Python OpenCV library. The shape estimation and conversion to robot trajectory steps are shown in Fig. 1.

### Experiments

The experimental setup is shown in Fig. 2d) and comprises of single RGB camera (Logitech International SA, Lausanne, Switzerland) positioned with a birds-eye view, normal to the profile of the object of which shape we want to detect. Three datasets of images were obtained to test the accuracy of the shape sensor.

1. Colourbands inside their moulds Fig. 2a))
2. Colourbands on the staircase (Fig. 2b))
3. Rails sunctioned to the staircase (Fig. 2c))

In each of these datasets, images were acquired from the top-down view. The camera was manually positioned to ensure it was parallel to the scene. To assess the accuracy of the vision-based shape sensor compared with data we obtained from the FBGS-based sensing in our previous paper [10], we manufactured five green-coloured silicone bands in DragonSkin^TM^ 30 silicone elastomer (shore hardness 30 A) (Smooth-On Inc., Macungie, PA, US), as shown in Fig. 2a). These colourbands were left in their moulds to ensure they were in the correct shape. In the first experiment, ten images were taken of each of the bands in the experimental setup shown in Fig. 2a). The shape sensing was performed as outlined in Sect. 2.2 to get a series of 2D coordinate points.

Then, the bands were removed from their moulds and placed on the staircase, as shown in Fig. 2b) and the image acquisition was repeated.

Then, the PAF rail was suctioned to each of the curved profiles of the staircase, as shown in Fig. 2c) and the image acquisition was repeated. The data obtained from the shape sensor was a set of experimental curves *Y* in the form of a 2D array. To perform a meaningful comparison between the experimental data *Y* and the ground truth curves *X*, obtained from the CAD model of the staircase, procrustes alignment was performed. The outcome of this process aligns the two coordinate frames of the ground truth curves and the experimental ones. Root mean square error (RMSE) was employed to quantify the difference between the aligned curves.

**Fig. 3:**
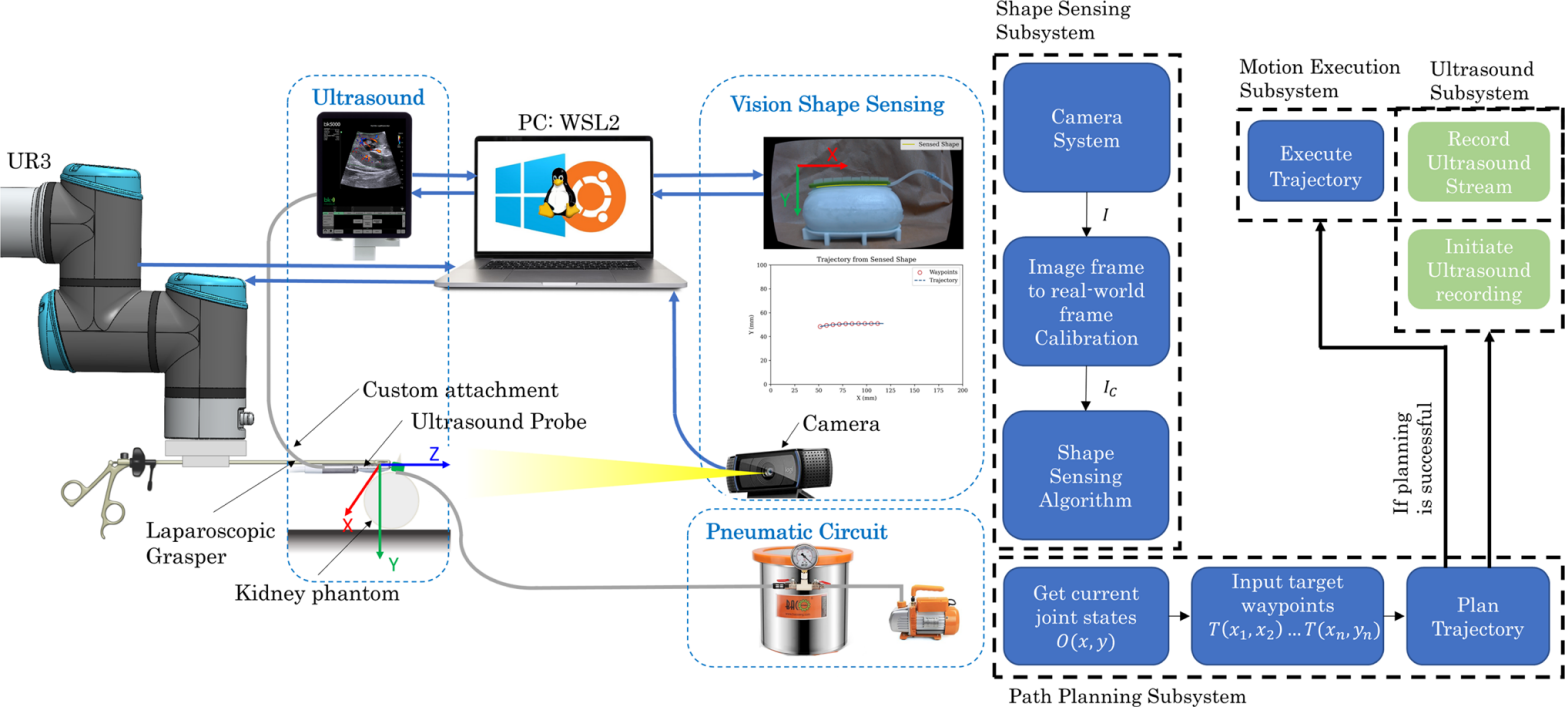
Schematic diagram of the setup for robot-acquired US images. The system comprises of three main subsystems; the vision shape-sensing subsystem, the US subsystem and the robot subsystem. The US cart and Universal Robots UR3 robot are connected to the computer using ethernet cables. The camera is connected to the computer via USB. The PAF rail is attached to a kidney phantom using pneumatic suction

### Robot control

To facilitate proof-of-concept experiments, we used a Universal Robots UR3 (Universal Robots, Odense, Denmark). The shape sensing aims to obtain a set of 2D coordinates that represent the shape of the PAF rail so that this can be converted into a trajectory for robot arm to execute while gripping the US probe. In doing so, the robot should be able to acquire US images autonomously.

We equipped the UR3 end effector with a hand-held laparoscopic grasping tool (Ruihui Electronic Technology Co. Ltd., Zhengzhou, China), by printing a custom attachment, designed in SolidWorks (Dassault Systèmes, Vélizy-Villacoublay, France) and printed using a FormLabs Form 3 (Somerville, MA, US) printed in Tough 2000 resin. The grasping tool was then fixed to grasp an X12C4 BK Robotic Drop-In US Transducer connected to a BK5000 Ultrasound cart (both by BK-Medical Holding Inc., Peabody, Massachusetts) for US image acquisition. The US probe is encased in a custom 3D-printed mount as introduced in [29]. This allows it to be grasped by the grasp tool while also being connected and able to roll along the PAF rail.

We communicated with the robot using robotic operating system 2 (ROS2) installed on Windows Subsystem for Linux 2 (WSL2), such that the shape-sensing application, robot control and US image acquisition could be performed simultaneously. The robot is controlled using the MoveIt open motion planning library (OMPL). A full schematic of the experimental setup and the scheme of the control system for the robot is depicted in Fig. 3.

**Fig. 4:**
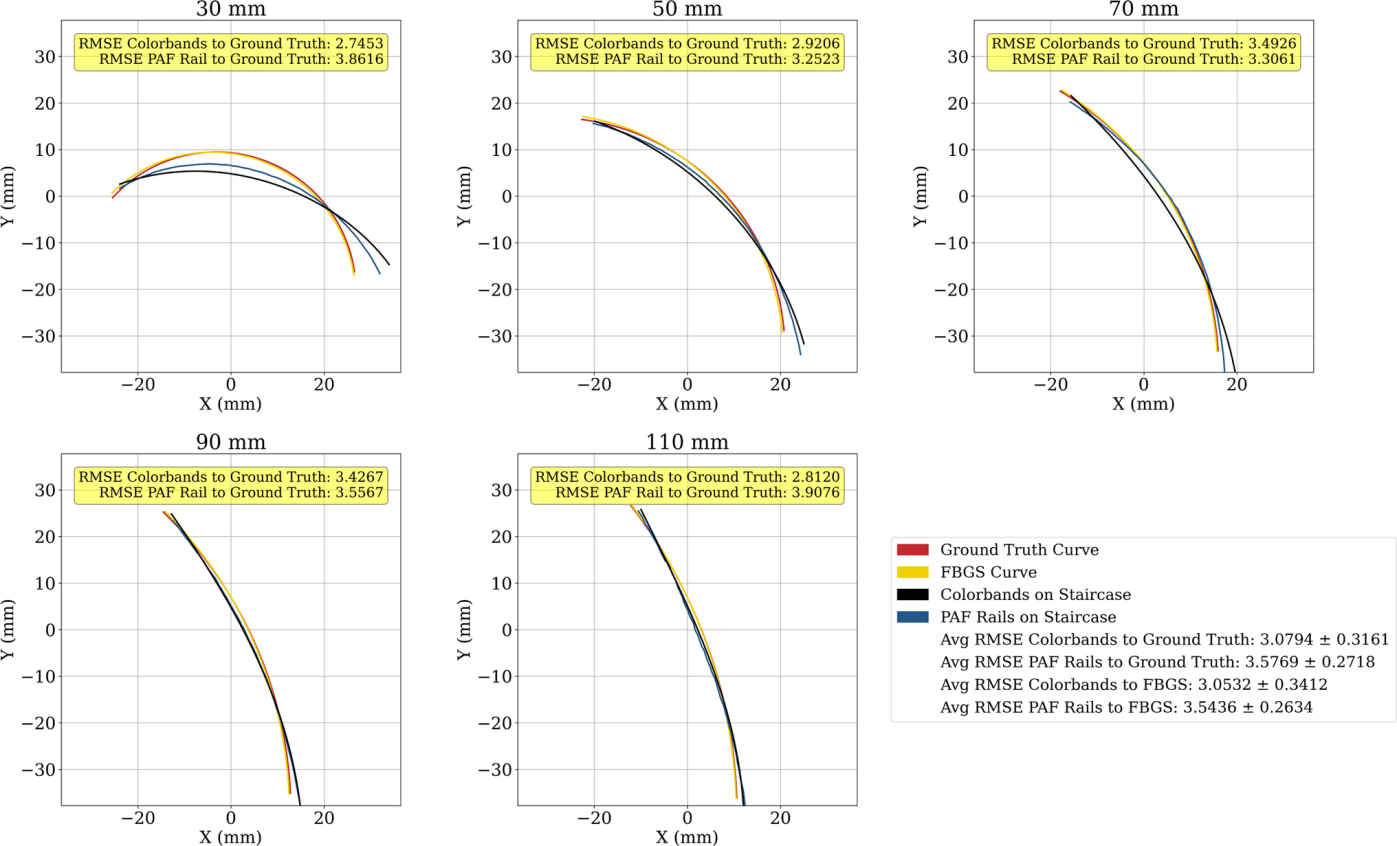
Plots show ground truth geometric curve (red) for each of the radii tested (30 to 110 mm) compared with the FBGS curve (yellow) and experimental curves obtained by vision- based shape sensing of the colourbands (black) and PAF rails (blue) suctioned to the curved profiles of the staircase. Pointwise RMSE between the experimental data and ground truth is calculated for each of the radii

We simplified the shape-sensing problem to two dimensions: *x* and *y* as the motion of the probe is constrained in the *z* direction due to the pairing with the PAF rail. In the real-world frame, we achieved this by manually positioning the US probe at one end of the PAF rail by connecting the roller to it.

In real time and online, we performed the shape sensing on the PAF rail suctioned to a kidney phantom. These coordinates were then published to a ROS2 topic. A ROS2 package was created containing a node which subscribed to the coordinate topic and node which subscribed to the output robot pose of the robot. The UR3 executed the trajectory at 20$\%$ of its maximum velocity to replicate the clinical scenario most closely. The US stream was recorded simultaneously using the Scikit-SurgeryBK library [30] and saved as a.mp4 file. The frame rate of the system is 25 frames per second.

The end effector pose was recorded by subscribing to the ROS2 robot pose publisher node. We extracted the executed trajectory from the *x* and *y* coordinates of the pose of the end effector joint. We validated the system by comparing freehand US acquired images of the phantom with those obtained by the UR3-controlled probe. Seven freehand US videos were obtained by manually moving the US probe along the rail. Two clinicians of varied experience were asked to review 22 US videos. The videos were ordered randomly, and reviewers were blind to whether a human or robot acquired the US videos. They were asked to comment by rating on a 5-point Likert scale (Excellent, Good, Fair, Poor, Very Poor), on three criteria:

1. *RES* Sharpness and crispness of image, and a lack of haziness/blurriness.
2. *DET* Clarity of structure outlines/ease of which boundaries are seen.
3. *IQ* Contrast of solid and fluid-filled structures and absence of noise.

These criteria were based on those in [31]. It must be noted that this comparison is subjective and that US evaluation is highly dependent on how it is acquired and for what purpose.

**Fig. 5:**
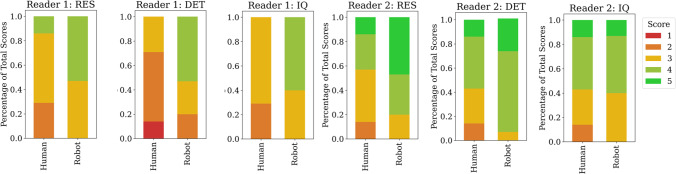
Distribution of Likert scores assigned to each of the 22 ultrasound videos from reader 1 (left) and reader 2 (right), for the following criteria: RES, DET and IQ

## Results and discussion

### Colour-based shape estimation

As a first step, we wanted to evaluate the vision-based shape sensor. To do so, we tested the system on coloured curves of known radius, held in moulds to ensure their shape. Then, we plotted the sensed shape of the colourbands against the known geometrical curve to visualise the accuracy of the shape-sensing system.

We evaluated the vision-based shape sensor on the staircase described in Sect. 2.3 to provide comparison data for the PAF rail. The results of this are shown in Fig. 4. The average pointwise RMSE between the vision-sensed curve of colourbands placed on the staircase and geometric ground truth is $3.0794 \pm 0.3161$ mm. We attribute this discrepancy to the fact that in practice, it is difficult to perfectly align the curve of the colourbands with the profile of staircase.

Finally, we evaluated the vision-based shape sensing on the PAF rail suctioned to the staircase. The results of this are shown in Fig. 4. We found the average pointwise RMSE between the vision-sensed curve of the PAF rail and geometric ground truth to be $3.5769 \pm 0.2718$ mm. The difference in RMSE between vision-sensed colourbands and vision-sensed PAF rail is $0.4975 \pm 0.4169$ mm, indicating sub-millimetre accuracy of the vision-based sensing system. The largest source of error compared to ground truth occurs at $r=30 mm$. As seen in Fig. 4, the sensed curve of the PAF rail has a greater radius than ground truth FBGS. The suction cup depth of the PAF rail is 4 mm, introducing an offset error that is not systematic due to the way in which the suction cup deforms on profiles of different radius. In addition, at smaller radii, the PAF rail is under greater strain to facilitate suction, and deforms tangentially rather than concentrically with the profile it is suctioned to. Therefore, this large error to geometric ground truth is attributed to the deformation of the PAF rail and not the sensing system. Omitting the 30 mm data, the average pointwise RMSE between vision-sensed curve of the PAF rail and geometric ground truth becomes $3.505 \pm 0.1878$ mm, representing an average sensing error of $4.7 \%$ relative to the measured radii. While the magnitude of the error remains somewhat the same over each of the radii measured, the significance of this error is smaller for larger radii. Given that the curvature of the kidney surface where the PAF rail is suctioned during RAPN is more likely to be closer to 110 mm, we believe that this RMSE is therefore sufficient for sensing the shape of the PAF rail.

### Robot control

Using the scheme depicted in Fig. 3, 20 trajectories were executed using coordinate information obtained from the vision-based sensor. As such, 20 US streams were recorded at a frame rate of 25 frames per second (fps).

Of these 20 US acquisitions, 15 had continuous visualisation of the kidney phantom, indicating adequate contact between the US element and phantom for imaging. Of the 5 that did not contain a continuous image, despite the successful execution of the planned trajectory, the US element either lost contact or did not apply enough pressure for imaging. The custom attachment that pairs the US probe with the PAF rail allows translational movement along the PAF rail and pivoting around the rail’s axis for consistent contact. This small amount of rotational freedom caused the probe to lift off the organ’s surface during certain paths. To address this, future work will involve using stereo cameras for 3D depth information to enhance path planning and incorporating force sensing to provide feedback on probe contact with the imaging target.

### Clinical evaluation of ultrasound

For clinical evaluation, the US videos were pre-sorted by a non-expert as to whether they contained a continuous image stream. As described in Sect. 3.2, five videos were discarded, leaving 15 for clinical evaluation. As well as this, seven freehand US videos were obtained by manually moving the ultrasound probe along the rail by a non-expert. The scoring results from each reader are shown in Fig. 5. The scores are colour-coded using a traffic light colour scheme to indicate 1 as ‘Very Poor’ and 5 as ‘Very Good’. For both reader 1 and reader 2, across all criteria, the median score for human was 3—‘Good’, and the median score for robot was 4—‘Very Good’. This figure indicates that, at a minimum, all images acquired were of good enough quality for clinical evaluation. In particular, clinicians noted that the resolution of the robot-acquired images was superior to the human-acquired ones, with 100 % of RES scores assigned to at least ‘Good’ by both readers. The full breakdown and frequency of scores allocated are shown in Table I and Table II in the supplementary material. The key result here is that two independent clinical interpreters regarded the robot-acquired US images to be at least as good as the freehand-acquired ones.

## Conclusion and future work

We propose a vision-based system for autonomous intra-operative US scanning, using colour image segmentation with OpenCV. This low-cost framework, adaptable to various soft interfaces and robotic devices, estimates the 2D shape of a flexible interface. We tested the system against coloured curves between $R = 30$ mm and $R = 110$ mm, finding the vision system’s average error to be $3.0794 \pm 0.3161$ mm. We evaluated the system against the PAF rail on the same curves and found an average error of $0.4975 \pm 0.4169$ mm. We show that this precision proves adequate for robotic control. In real time, our method informs a UR3 robot to follow the desired path of the PAF rail on an organ phantom. Paired with an intra-operative ultrasound (IOUS) probe, the robot performs consistent US scans. We validate this by obtaining expert clinical evaluation of the obtained US images. The clinicians concurred that the robot-acquired images were of at least ‘Good’ in all quality metrics and outperformed the human-acquired images in all quality metrics.

In future work, we will translate this framework to a da Vinci surgical robot paired with the da Vinci Research Kit (dVRK). We aim to further quantitatively evaluate the US image acquisition process and resultant US images with clinical experts.

### Supplementary Information

Below is the link to the electronic supplementary material.

Supplementary file 1 (mp4 18889 KB)

Supplementary file 2 (pdf 37 KB)
