# Supplementary material for: Autonomous control of an ultrasound probe for intra-operative ultrasonography using vision-based shape sensing of pneumatically attachable flexible rails
Source: Int J Comput Assist Radiol Surg. 2024 May 22;19(7):1391–8. doi: 10.1007/s11548-024-03178-z (PMC11230978; doi:10.1007/s11548-024-03178-z)
Supplement: Supplementary file 2 — (pdf 37 KB) [file 11548_2024_3178_MOESM2_ESM.pdf]

Vision-based Shape Sensing of Pneumatically Attachable Flexible Rails for Autonomous Control of an Ultrasound Probe for Intra-Operative Ultrasonography: Supplementary Material

Aoife McDonald-Bowyer[1] *Student Member, IEEE*, Tom Syer[2], Adam Retter[3], Danail Stoyanov[1] *Senior Member, IEEE*, Agostino Stilli[1] *Member, IEEE*

TABLE I

Criteria		Reader 1: Score(%)				
		1	2	3	4	5
SEC	Human	1 (14%)	4 (57%)	2 (29%)	0 (0%)	0 (0%)
	Robot	0 (0%)	2 (29%)	4 (57%)	1 (14%)	0 (0%)
RES	Human	0 (0%)	3 (20%)	4 (27%)	8 (53%)	0 (0%)
	Robot	0 (0%)	0 (0%)	7 (47%)	8 (53%)	0 (0%)
IQ	Human	0 (0%)	2 (29%)	5 (71%)	0 (0%)	0 (0%)
	Robot	0 (0%)	0 (0%)	6 (40%)	9 (60%)	0 (0%)

TABLE II

Criteria		Reader 2: Score(%)				
		1	2	3	4	5
SEC	Human	0 (0%)	1 (14%)	2 (29%)	3 (43%)	1 (14%)
	Robot	0 (0%)	1 (14%)	3 (43%)	2 (29%)	1 (14%)
RES	Human	0 (0%)	0 (0%)	1 (7%)	10 (67%)	4 (27%)
	Robot	0 (0%)	0 (0%)	3 (20%)	5 (33%)	7 (47%)
IQ	Human	0 (0%)	1 (14%)	2 (29%)	3 (43%)	1 (14%)
	Robot	0 (0%)	0 (0%)	6 (40%)	7 (47%)	2 (13%)
